# Derivation of a novel antimicrobial peptide from the Red Sea Brine Pools modified to enhance its anticancer activity against U2OS cells

**DOI:** 10.1186/s12896-024-00835-8

**Published:** 2024-03-15

**Authors:** Mona Elradi, Ahmed I. Ahmed, Ahmed M. Saleh, Khaled M. A. Abdel-Raouf, Lina Berika, Yara Daoud, Asma Amleh

**Affiliations:** 1 Biotechnology Program, American University in Cairo, New Cairo, Egypt; 2 Biology Department, American University in Cairo, New Cairo, Egypt

**Keywords:** Anticancer peptides, Antimicrobial peptides, Red Sea Metagenomic Library, Osteosarcoma, Apoptosis

## Abstract

Cancer associated drug resistance is a major cause for cancer aggravation, particularly as conventional therapies have presented limited efficiency, low specificity, resulting in long term deleterious side effects. Peptide based drugs have emerged as potential alternative cancer treatment tools due to their selectivity, ease of design and synthesis, safety profile, and low cost of manufacturing. In this study, we utilized the Red Sea metagenomics database, generated during AUC/KAUST Red Sea microbiome project, to derive a viable anticancer peptide (ACP). We generated a set of peptide hits from our library that shared similar composition to ACPs. A peptide with a homeodomain was selected, modified to improve its anticancer properties, verified to maintain high anticancer properties, and processed for further *in-silico* prediction of structure and function. The peptide’s anticancer properties were then assessed in vitro on osteosarcoma U2OS cells, through cytotoxicity assay (MTT assay), scratch-wound healing assay, apoptosis/necrosis detection assay (Annexin/PI assay), RNA expression analysis of Caspase 3, KI67 and Survivin, and protein expression of PARP1. L929 mouse fibroblasts were also assessed for cytotoxicity treatment. In addition, the antimicrobial activity of the peptide was also examined on *E coli* and *S. aureus*, as sample representative species of the human bacterial microbiome, by examining viability, disk diffusion, morphological assessment, and hemolytic analysis. We observed a dose dependent cytotoxic response from peptide treatment of U2OS, with a higher tolerance in L929s. Wound closure was debilitated in cells exposed to the peptide, while annexin fluorescent imaging suggested peptide treatment caused apoptosis as a major mode of cell death. Caspase 3 gene expression was not altered, while KI67 and Survivin were both downregulated in peptide treated cells. Additionally, PARP-1 protein analysis showed a decrease in expression with peptide exposure. The peptide exhibited minimal antimicrobial activity on critical human microbiome species *E. coli* and *S. aureus*, with a low inhibition rate, maintenance of structural morphology and minimal hemolytic impact. These findings suggest our novel peptide displayed preliminary ACP properties against U2OS cells, through limited specificity, while triggering apoptosis as a primary mode of cell death and while having minimal impact on the microbiological species *E. coli* and *S. aureus*.

**Supplementary Information:**

The online version contains supplementary material available at 10.1186/s12896-024-00835-8.

## Introduction

Cancer is one of the leading causes of death worldwide and is estimated to surpass cardiovascular diseases in two decades [1]. The limited efficiency of current conventional onco-therapeutic options, together with emerging resistance of cancer cells to a wide range of chemotherapeutic drugs, adds to the burden of cancer [2]. Protein based drugs, or protein biologics, such as insulin and monoclonal antibodies, were established with advancements in molecular biology tools and recombinant protein expression technology. Large protein size (> 5 kDa) provides increased specificity for the target antigens at the cost of adequate membrane penetration, affordable synthesis, bioavailability, and metabolic stability [3]. Alternatively, small scale peptide-based drugs (< 0.5 kDa) that have a biological equivalent in antimicrobial peptides (AMPs) display high specificity to their molecular targets, more membrane penetration, a less immunogenic response and overall fewer side effects [3–5]. AMPs are a component of the innate immunity in almost all eukaryotes and possess the ability to disrupt bacterial membranes based on biophysical properties such as their amphipathicity, hydrophobicity, size, secondary structure, and most importantly the non-specific electrostatic interaction with the anionic lipid rich microbial membrane [6].

Anticancer peptides (ACPs) exert their cytotoxic activities in a similar mechanism to AMPs as they share similar biophysical properties attributed to their analogous synthesis origins and their targeting of relatively negative charged tumor and bacterial cells [7]. The ability for ACPs to selectively target cancer cells is still being elucidated and researchers seek to understand the mechanistic actions by which cancer cellular death is actuated. Kinetics associated with cancer cell death have been shown to involve membranolytic and non-membranolytic modes of action [8]. Membranolysis involves mitochondrial membrane disruption to release initiators of apoptosis which can explain the late death events with some ACP examples [9]. Membrane disruption occurs through either pore formation (toroidal and barrel stave model), thinning and membrane dissolution (detergent-like Carpet model), lipid peptide domain formation or membrane depolarization [10]. However, non-receptor mediated membrane lysis events still best describe the rapid and selective ACP induced cytotoxicity [11]. Additionally structural analysis of those peptides with α-helical conformations were associated with membranolytic anticancer activity [12–14], and when combined with hydrophobic properties, enhance membrane permeabilization, aid nuclear absorption and the initiation of an apoptotic cascade [2, 15].

Given the similar mechanisms of action between ACP and AMPs, and in an effort to offer better cancer therapies, we aimed to identify sequence hits corresponding to a peptide that showed composition similarity to known experimentally verified ACPs by utilizing the unexplored Red Sea metagenomics library, created during AUC/KAUST Red Sea expeditions in 2008 & 2010. This database offers a novel bacterial microenvironment dataset [16]. The selected peptide was computationally analyzed to determine structural and functional predictions while adjusting the hydrophobicity, cataionicity, and size of the peptide to improve its anticancer properties. We validated the *in-silico* findings through in vitro testing of the peptide on the Osteosarcoma cell line U2OS by examining cell cytotoxicity and comparing it to L929 mouse fibroblasts. U2OS were also examined for morphological features when treated with multiple concentrations of the peptide. The mode of cell death was interrogated via apoptosis and necrosis detection assay, RNA expression analysis of Caspase 3, and PARP-1 protein expression. Additionally, gene expression of KI67 and Survivin were also assessed to determine the effect of the peptide on proliferation and inhibition markers, respectively. In addition, the antimicrobial activity of the peptide was examined to assess its cytotoxicity at the microbiological level on two model species, *Escherichia coli* and *Staphylococcus aureus*, bacterial species prominent in the human microbiome.

## Materials and methods

### Computational analysis

#### Red sea metagenomics library scanning

The raw data collection method, quality control measures, adaptive Support Vector Machine model, and screening of the metagenomic library was established based on previous work [17]. Briefly, the scanning process of the Metagenomic library was conducted via a previously described method [18] where both ACPs and non-ACPs derived from AMPs were recruited. A set of experimentally validated ACPs was acquired from publicly available AMP databases, namely, APD and APD2 [19, 20], the collection of antimicrobial peptides CAMP [21] and the database of Anuran defense peptides DADP [22]. An additional set of peptides not designated with any anticancer activity was recruited from these databases. Single and double amino acid (aa) compositions were used as input features when comparing the two datasets in selecting only those peptides recognized as ACPs (t- test, *p* < 0.05, Bonferroni multiple testing correction). Metagenomic reads were aggregated through a sliding window of increasing peptide size (minimum 5 aa) to generate stretches of peptide sequences. A total of 59 peptide sequences were examined for Hidden Markov Models (HMMs) with various scores and lengths [23], and cross-referenced with experimentally validated ACPs. These were fed into an online tool, "AntiCP" (Institute of microbial technology, Chandigarh, India), for ACP sequence status verification [18]. A 30 aa homeodomain sequence (PF00046.24) was selected from the total set of 59 peptides. A four aa sequence addition, consisting of 2 Alanine, Glutamate, and a Lysine, (AAEK) was appended to the start of the peptide sequence, outside the HMM domain, to increase its hydrophobicity and charge, and overall penetrability. The newly 34 aa amalgam sequence (AAEKEFIKYPYPTPLQYQQLATRLKVEKKLVRRW) was verified as an ACP through the AntiCP webserver and was aligned against the same AMP and ACP datasets, in addition to alignment against the non-redundant protein dataset in PDB and SwissProt, via the Blastp webtool [24].

#### *In-silico* predictions of the sequence

The half-life of the peptide sequence was first determined in an intestine-like environment with HLP [25]. Additionally, the PlifePred webserver was utilized to predict half-life of the blood-borne peptide residual [26]. The sequence was also examined for potential toxicity with Toxinpred, offering more than 2000 experimentally tested toxic peptides to contrast [27]. The peptide sequence was aligned against the ApoCanD dataset of human apoptotic proteins associated with cancer [28]. The 3D structure of the peptide sequence was predicted through the I-Tasser, which also provided comparable templates of other sequences with high structural homology [14, 29]. Additionally, COACH & COFACTOR tools, from the same webserver, provided a prediction for the ligand binding site & biological function annotation of the peptide, respectively.

#### *In-silico* analysis of the antimicrobial activity

The antimicrobial activity of the peptide was computationally assessed against *E. coli* (ATCC 25922) and *S. aureus* (ATCC 25923) strains using the PAASS tool on DBAASP v3 webserver [30]. Peptides classified as not active were associated with negative predictive values (NPVs) indicative of probability substrate will be inactive against the species of interest. Furthermore, AMPA tool was used to assess the antimicrobial activity of each region of the peptide’s sequence [31].

### In vitro analysis

#### Synthesis of peptide

The peptide was synthesized by FlexPeptide™ technology (Genscript, USA), utilizing high performance liquid chromatography (HPLC) for purification. The peptide (> 95% purity) was received in aliquots of a lyophilized powder, was subsequently dissolved, and sterilely filtered in phosphate buffered solution (PBS). The synthesized peptide had these characteristics: Chemical formula: C197H312N52O49; Molecular weight: 4192.92 Da; Charge: + 5 (Cationic).

#### Cell culture

Osteosarcoma cells U2OS and normal mouse fibroblast cells L929 were a gift from Dr. Andreas Kakarougkas, American University in Cairo (AUC). Cells were cultured in DMEM (Dulbecco’s Modified Eagle Medium) (Gibco, Paisley, Scotland, UK) supplemented with 10% fetal bovine serum (FBS) (Gibco, Paisley, Scotland, UK) and 5% Penicillin–streptomycin antibiotic (Gibco, Paisley, Scotland, UK). Cells were incubated in a CO_2_ incubator (Thermo Fisher Scientific, Vilnius, Vilniaus County, Lithuania) at 37ºC and 5% CO_2_, where they were regularly split at 70–80% confluence following a wash with phosphate buffer saline (PBS) and enzymatic detachment via Trypsin–EDTA (Gibco, Paisley, Scotland, UK). Cells were examined using an inverted microscope (Olympus IX70, Essex, England, UK) and stained with Trypan blue (Gibco, Paisley, Scotland, UK) for counting via a hemocytometer (Hausser Scientific, USA).

#### Cytotoxicity MTT assay

U2OS cells were seeded in 96-well plates (Greiner Bio-one, Frickenhausen, Baden-Württemberg, Germany) at 1 × 10^4^ cells/well, and were incubated for 24 h following seeding. Serial concentrations of the peptide were diluted in fresh media to 1, 10, 100, 300, and 500 µg/ml and used to treat cells for a further 24 h. A different concentration gradient was tested for L929 cells (0.01, 0.1, 1, 10, and 100 µg/ml). MTT (Serva, Heidelberg Baden-Württemberg, Germany) reagent was dissolved in fresh media at 1 mg/ml and incubated for 3—4 h away from a light source. Crystalline formations were solubilized via exposure to Dimethyl sulfoxide (DMSO) (Sigma-Aldrich, St. Louis, MO, USA). Absorbance values were measured at 492 nm using a microplate reader FLUOstar OPTIMA (BMG LabTech, Ortenberg, Baden-Württemberg, Germany). IC50 values were calculated in GraphPad Prism 7.0 via analysis of blank corrected absorbance values, where untreated cell absorbance represented 100% viability.

#### Scratch wound closure assay

Scratch wound closure assay was conducted to test the effect of the peptide treatment on the capacity for U2OS cells to migrate. Cells were cultured and seeded at 1 × 10^5^ in 24 well plates. Scratch-wounds were performed following 80% confluency, by pressing a sterile 20 μl pipette tip across each well to produce two perpendicular lines. Cells were subsequently washed twice with 1X PBS. Growth media was added to the untreated control, while media supplemented with IC50 of the peptide and added to the treated wells and incubated for 24 h. Scratch-wounds were imaged at time points 0 h and 24 h, with an Olympus IX70 inverted microscope.

#### Annexin V/ propidium iodide assay

Annexin V/Propidium Iodide (PI) assay was performed according to manufacturer's protocol (Thermo Fisher Scientific, Vilnius, Vilniaus County, Lithuania) and adapted for imaging fluorescent microscopy (Olympus IX70, USA). Cell death was interrogated by incubating 33 × 10^4^ U2OS cells/well in a 6 well plate overnight, exposing them in 200 µg/ml of peptide for 24 h. Cells were subsequently trypsinized, while supernatant media was collected containing floating cells and stained with 10 µl Annexin V conjugate (Alexa fluor 488) and 1 µl of PI working solution. Cells were left at room temperature away from light interference for 15 – 20 min, and subsequently visualized under the fluorescent microscope at 488 nm and 617 nm.

#### Reverse transcription-PCR

Total RNA was extracted using TRIzol (Thermo Fisher Scientific, Vilnius, Vilniaus County, Lithuania) as per the manufacturer's recommendations. Peptide treatment (IC50; 100.5 µg/ml) of U2OS cells and untreated cells were examined for gene expression. cDNA synthesis was conducted with defined RNA concentrations, using the Revert ID™ First Strand cDNA synthesis kit (Fermentas, Hanover, Maryland, USA). PCR reactions were guided by combining 1 µg of cDNA with Taq DNA Polymerase (Thermo Fisher Scientific, Vilnius, Vilniaus County, Lithuania), and gene specific primers, in optimized reaction conditions. Caspase 3 primers consisted of forward primer (GACCATACATGGGAGCAAGT) reverse primer (ATCCGTACCAGAGCGAGA). GAPDH forward primer: (CCACCCATGGCAAATTCCATGGCA) and reverse primer:(TCTAGACGGCAGGTCAGGTCCACC). KI67 forward primer: (CTTGGGTGCGACTTGACG) and reverse primer: (GTCGACCCGCTCCTTTT). Survivin primers consisted of forward: (ATGGGTGCCCGACGTTG) and reverse: (GGCCAGAGGCCTCAATCCAT).

#### Western blot expression

Protein extraction was conducted via TRIzol according to the manufacturer's recommendations. Polyacrylamide gels were prepared by combining 40% acryl to 2% Bis, 1 M Tris pH 8.7, 1 M Tris pH 6.9, 20% SDS and water (All Sigma-Aldrich, St. Louis, MO, USA). TEMED (Sigma-Aldrich, St. Louis, MO, USA) and 10% Ammonium Persulfate (Serva, Heidelberg Baden-Württemberg, Germany) were added immediately prior to gel casting. Gels were run at 150 V for 90 min and blotted on a Nitrocellulose membrane (Thermo Fisher Scientific, Vilnius, Vilniaus County, Lithuania) at 150 V for 60 min. Membranes were blocked in 5% skimmed milk Tris Buffer Saline (TBS) solution with 1% Tween (Serva, Heidelberg Baden-Württemberg, Germany) blocking solution for 1 h. Membrane was then soaked overnight in primary antibody PARP (46D11) (mAb #9532, Cell Signaling Technologies, USA) in blocking solution on a rocker. Membranes were washed with TBS Tween and subject to goat anti-rabbit secondary antibodies (Thermo Fisher Scientific, Vilnius, Vilniaus County, Lithuania) in blocking solution for 2 h, washed with TBS Tween, followed by BCPI/NBT phosphatase substrate (Serva, Heidelberg Baden-Württemberg, Germany) detection.

#### Disk diffusion assay

Overnight cultures of *S. aureus* and *E. coli* were diluted at 1 × 10^5^ Colony Forming Units (CFUs)/ml and spread homogenously on agar plates. Filter paper disks (6 mm) were impregnated with the IC50 concentration of the peptide and placed on agar plates. Clindamycin (2 µg) and Erythromycin (15 µg) antibiotics were used as positive controls for the *S. aureus* agar plates while Imipenem (10 µg) and Tetracycline (30 µg) antibiotics were used as positive controls for the *E. coli* agar plates. Plates were incubated for 24 h at 37 ºC and diameters of the corresponding inhibition zones were measured.

#### Antimicrobial sensitivity

Bacterial inoculant glycerol stock of *S. aureus* (ATCC 6538) and *E. coli* DH5-alpha (ATCC PTA-4752) were dissolved in freshly prepared LB broth (Sigma-Aldrich, St. Louis, MO, USA) where stock was dissolved in media and incubated at 37 ºC overnight in a shaking incubator. Overnight cultures were then serially diluted by a factor of 1 × 10^5^ CFU/ml and IC50 concentration of the peptide (based on U2OS IC50 calculated estimate) was added to fresh broth. Culture was spread on LB agar (Sigma-Aldrich, St. Louis, MO, USA) plates and incubated at 37 ºC overnight. Colonies were counted the next day and CFU/ml were calculated.

#### Scanning electron microscopy

*S. aureus* and *E. coli* cultures were serially diluted to a factor of 2 × 10^6^ CFU/ml, then treated with the IC50 of the peptide for 1 h at 37 °C. Ampicillin-treated (10 µg) samples and untreated samples were used as positive and negative controls, respectively. Cells were fixed using 2.5% glutaraldehyde, dehydrated with 35%, 50%, 75%, 95%, and 100% EtOH, and coated using gold sputtering with HUMMER 8 at 10 mA for 1 min. Cells were imaged using a LEO Supra 55 FE-Scanning Electron Microscope (SEM), at two magnification powers ranges: lower range imaging (1000x – 1790x) and higher range imaging (2000x – 7760x) to observe colony formation and cell morphology.

#### Hemolytic activity assay

Acquisition of human blood was mediated by approval from an internal Institutional Research Board at AUC. Three milliliters of blood were collected, where RBCs were isolated by centrifuging vials for 10 min at 1500 RPM, and cellular precipitate was washed three times with 1%—2% saline solution then diluted tenfold in saline. Cells were diluted with equal volume of peptide IC50 solution and seeded on a 96-well plate for 1 h. Distilled water-treated samples were used as a positive control, saline-treated sample as a negative control. OD values were measured at 540 nm using a spectrophotometer.

#### Statistical data

Statistical and data analysis were performed using GraphPad Prism version 7.0. All results were presented as mean ± SD of three experimental replicas unless otherwise specified. Triplicate biological replicas were maintained for each condition per experiment, unless otherwise specified. An unpaired t-test was used for pairwise comparison between control samples and treated cell data. Results were deemed statistically significant at *P* < 0.05.

## Results

### In-Silico prediction

A list of 59 peptide sequences was identified, each with potential in-silico predicted anticancer activity. A 30 amino acid sequence peptide with a homeodomain HMM-ID was selected based on its potential anticancer attributes. The sequence was predicted as an ACP using the AntiCP webserver (Table 1) and confirmed to belong to a homeodomain protein family using Blastp (Table 2) and HMMER online webtools [18, 24]. Nearly all resulting significant matches were homeodomain proteins of different species. Homeodomain is a well-known family of transcription factors involved in embryogenesis, cell differentiation and in some instances, carcinogenesis [32]. The peptide sequence was analyzed for predictive half-life in an intestine-like environment, and was estimated to be nearly 1 s, with normal stability (Table 3), additionally, blood retention half-life was predicted to last 1373.41 s [25]. The I-Tasser and COACH web tools helped identify the peptide’s helical components, coiled regions, and determined a predictive nucleic acid binding site associated with the homeodomain sequence. Analysis of the predictive biological function of the peptide was done through the COFACTOR tool, where it was projected to have similar function to Antp (Antennapedia), a regulatory Drosophila homeoprotein involved in developmental regulatory pathways with enhanced penetrative properties [33]. This homeobox sequence has been structurally analyzed and functionally interrogated [34].

**Table 1:** Prediction of the peptide sequence as an ACP. The sequence was submitted to the AntiCP database and predicted to be an ACP before and after the addition of the newly introduced amino acids (AAEK). The sequence exhibits adequate amphipathic and hydrophobic properties of an ACP

Peptide Sequence	Mutation Position	SVM score	Prediction	Amphipathicity	Charge
AAEKEFIKYPYPTPLQYQQLATRLKVEKKLVRRW	No	0.69	Anticp	0.94	5

**Table 2:** Blastp alignment of the peptide sequence against a set of experimentally verified ACPs. It shows significant alignment with one ACP sequence from the Bacteriocin subgroup

Blastp Score	Expect	Method	Identities	Positives	Gaps
17.7 bits (34)	0.1	Compositional matrix adjust	8/17 (47%)	11/17 (64%)	0/17 (0%)

**Table 3:** Predicted half-life of the peptide. The HLP half-life prediction tool indicated a short half-life of the peptide sequence (around 1 s) with normal stability

Peptide Sequence	Mutation Position	Half-life (sec)	Stability	HPLC parameter	Hydrophobicity (KJ/mol)	pKa	pKb	Residue volume	Molecular weight	Isoelectric Point
AAEKEFIKYPYPTPLQYQQ LATRLKVEKKLVRRW	No	0.945	Normal	1.42	10.4	21.8	93.98	1481.5	1195.51	6.138

**Fig. 1:**
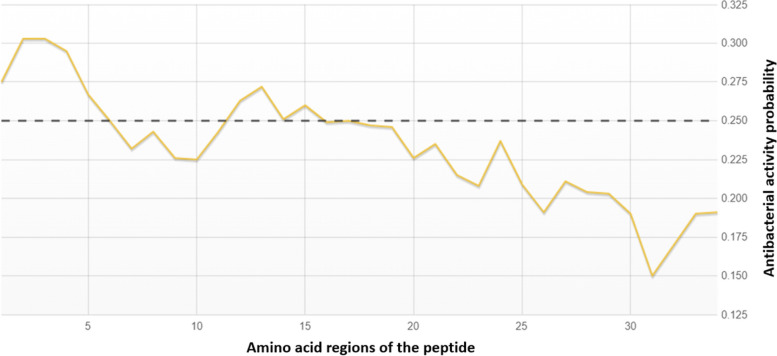
Prediction of the antimicrobial activity of the peptide by region. The graph shows the probability score of the antimicrobial activity of each 5 amino acids region of the peptide. The dashed line at 0.25 represents the default threshold of the antimicrobial activity by the tool

The antimicrobial activity of the peptide against *S. aureus* and *E. coli* species was classified by DBAASP to be non-active with 0.79 and 0.70 NPV scores, respectively, where 1.00 NPV corresponded to complete inactivity (Table 4) [30]. In addition, the antimicrobial predictor tool AMPA indicated that majority regions of the peptide had a low antimicrobial activity score (Fig. 1), below the default threshold, with the exception of sequences 1–6 and 12–16 amino acids [24]. These amino acid sequences displaying slightly higher antimicrobial activity, accounted for 32% of the entire peptide.

**Table 4:** DBAASP classification of the peptide’s antimicrobial activity. The peptide was tested against the *E. coli* and *S. aureus* strains recommended by the American Type Culture Collection and provided on DBAASP. The peptide exhibits no activity against both strains with a strong negative predictive value

ID	Strain Type	Class	Predictive Value (Type)
AMP_peptide	***Escherichia coli* ATCC 25922**	**Not Active**	**0.70 (NPV)**
AMP_peptide	***Staphylococcus aureus* ATCC 25923**	**Not Active**	**0.79 (NPV)**

### MTT cytotoxicity and cell morphology

**Fig. 2:**
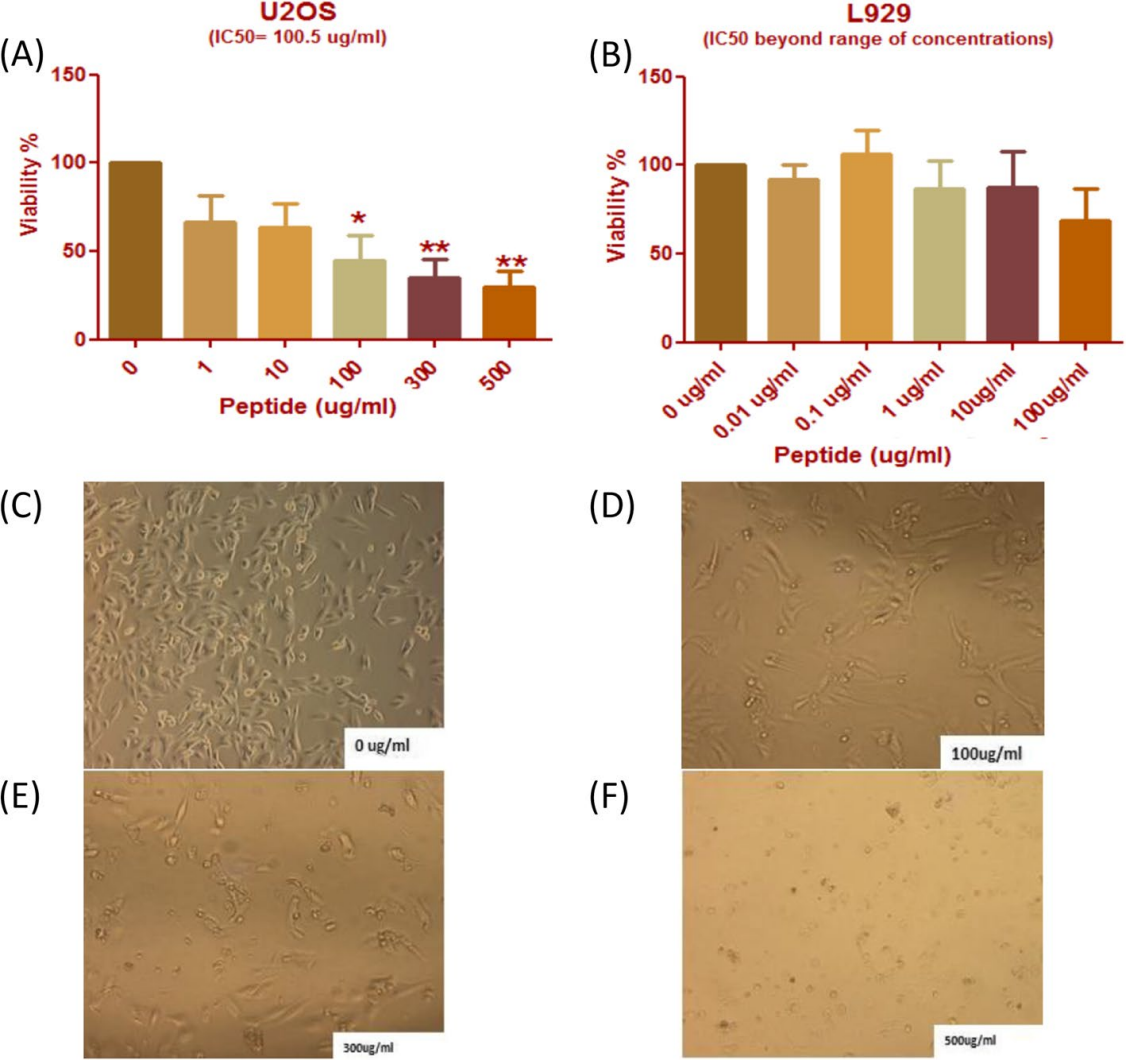
Dose dependent cytotoxicity of peptide treated U2OS and L929 cells, affects morphological changes. **A** Cells were treated with five concentrations of peptide (*n* = 8). Cells displayed a significant decrease in viability at 100 ug/ml and a greater decrease when exposed both 300 ug/ml and 500 ug/ml treatment (*P* < 0.05). IC50 value was calculated to be approximately 100.5 ug/ml. **B** No pattern of cytotoxicity could be observed with increasing concentrations of the peptide screening of L929 mouse cells. **C** Untreated cells displayed normal morphology of U2OS cells as fusiform in shape with intact membrane in 10 × magnification. **D** Cells treated with 100 ug/ml of peptide started to become more elongated and sparser, with shrunken volume. **E** At 300 ug/ml, there was a noticeable deformation appearance of the cells with lose of normal structure. **F** Treatment with 500 ug/ml of peptide resulted in cells lacking normal morphology, forming rounded cells with disrupted membranes

U2OS cells were exposed to ascending concentrations of peptide (Fig. 2A) for 24 h and showed a dose dependent cytotoxic effect. Cells displayed a significant decrease in cellular viability with exposure to 100 µg/ml, 300 µg/ml, and 500 µg/ml concentrations. The IC50 value was subsequently calculated and was determined to be approximately 100.5 µg/ml. L929 cells were treated to exponentially increasing concentrations of the peptide but did not show a variable response to treatment (Fig. 2B). No statistically significant differences were noted among treatment concentrations, and IC50 value was not computable.

U2OS cells were imaged to compare morphological variation in treated and untreated cells. Untreated cells displayed a fusiform spindle shaped morphology with intact membranes (Fig. 2C). Peptide- treated cells gradually changed morphology with increased concentration of the peptide. Cells exposed to 100 µg/ml were sparser and more elongated in structure and surface area (Fig. 2D). Treatment with 300 µg/ml caused some U2OS cells to lose their regular structure, while some maintained intact morphology (Fig. 2E). However, when screened with 500 µg/ml cells completely lost semblance of fusiform structure and formed rounded cells with disrupted membranes with a steady accumulation of debris in media (Fig. 2F).

### Scratch-wound healing assay

**Fig. 3:**
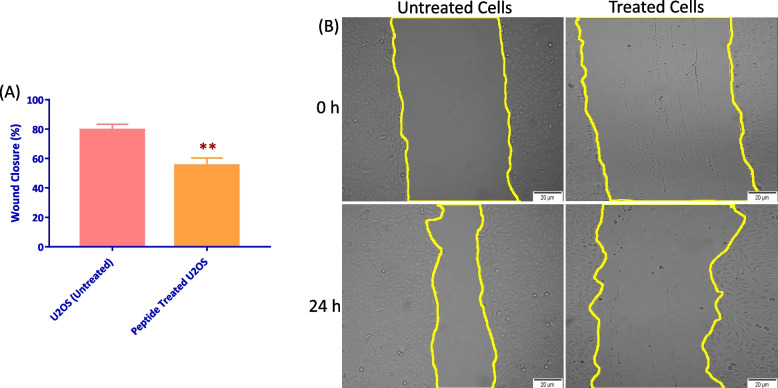
U2OS cells treated with IC50 concentration of the peptide showed reduced migration. U2OS cells were grown until sufficiently confluent and vertical scratches were administered and subsequently treated with IC50 of the peptide. **A** Wound closure percentage (%) of cells treated for 24 h. Closure decreased significantly, by approximately 20%, between peptide treated and untreated U2OS cells (*** *P* < 0.001, *n* = 6). **B** Wound closure representative images from untreated and treated U2OS cells with IC50 peptide for 24 h

U2OS cells were analyzed for migratory capacity via scratch-wound healing assay. A significant decrease in capabilities for cells to migrate was observed in treated wounds. Wound closure increased by 55% in cells treated with IC50 concentration of the peptide for 24 h, compared to 79% wound gap decrease with untreated cells (Fig. 3A). Representative images of scratch-wounds were indicative of the effect of the peptide compared to untreated cells, where control cells closed the scratch-wound gap significantly more than peptide treated U2OS cells (Fig. 3B).

### Annexin V/PI apoptosis assay

**Fig. 4:**
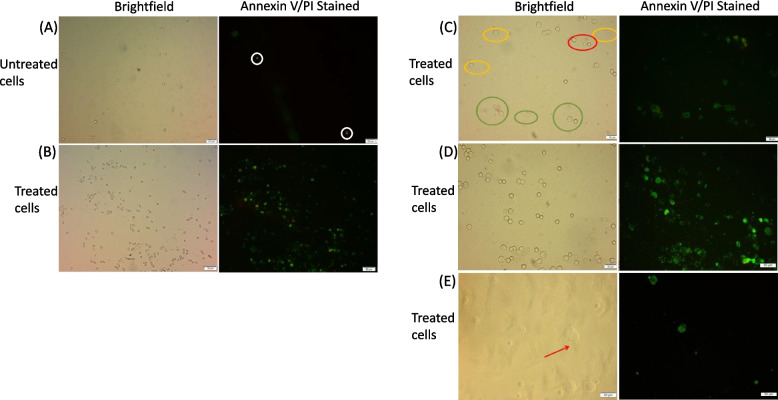
Annexin V assay of untreated and IC50 peptide treated U2OS cells. Brightfield and FITC Alexa fluor 488 channel microscopy. **A** Brightfield and stained untreated cell images (10 × magnifications) displayed a low number of dead cells (circled). **B** Peptide treated cells showed dominance of green stained cells indicating a progressive significant population of apoptotic population. **C** Yellow circular highlights were indicative of viable cells, green circular highlights were directive of apoptotic cells, while red circular highlights were prominent in apoptotic/necrotic cells. **D** Large scale apoptotic cell death was observed in highly confluent treated cells. **E** Arrow pointer towards budding formed by disrupted cells that is yet to be disrupted and uptake the stain

Cells were treated with Annexin/PI stains to elucidate mode of cell death resulting from peptide treatment. Untreated U2OS cells revealed a low number of green, red, and green/red stains as well as low fluorescence intensity (Fig. 4A). On the other hand, IC50 peptide treated U2OS cells exhibited high intensity green stained nuclei as a sign of early apoptosis (Fig. 4B). Red fluorescence was not detected alone without green fluorescence overlay. Higher magnification images (20x) highlighted distinctions between live/early apoptotic/necrotic cells (Fig. 4C). Early apoptosis indicators were specific to cells exposed to the peptide at higher resolution (Fig. 4D). Morphological changes were observed at 20 × magnification, where budding formations appeared in some cells, while others exhibited membrane disassembly into apoptotic bodies with no signal detection (Fig. 4E).

### Gene expression

**Fig. 5:**
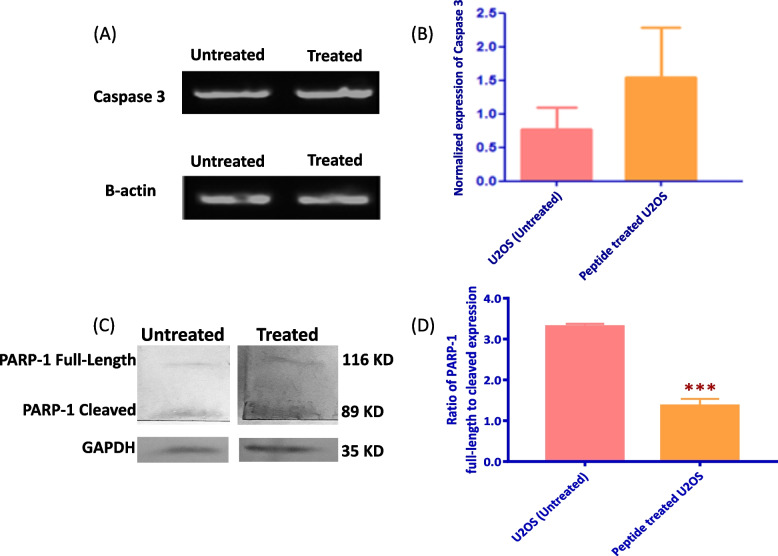
mRNA expression of Caspase 3 and protein expression of PARP-1 in untreated vs peptide treated U2OS cells and protein. **A** Gel electrophoresis expression images of Caspase 3 and B-actin (indigenous control) in treated and untreated cells. Untreated and treated samples of B-actin and Caspase 3 gel bands were run together on the same gel, respectively. Original unedited image of gel can be found in supplementary Fig. 5. **B** Normalized expression of Caspase 3 in untreated and treated cells shows no statistical change in expression (* *P* < 0.05, *n* = 6). **C** Membrane images of representative cleaved and full-length PARP-1 protein, and GAPDH expression in treated and untreated cells. Original unedited image of membrane can be found in supplementary Fig. 6. **D** Normalized expression levels of the ratio of PARP-1 full-length protein to cleaved protein. Treated cells showed a decrease in expression of the ratio of full-length PARP-1 protein to the cleaved version (*** *P* < 0.0001, *n* = 6)

U2OS cells were treated with IC50 concentration of the peptide for 24 h and were profiled for apoptotic marker Caspase 3, proliferation marker KI67, and caspase inhibitor Survivin [35, 36]. β-actin and GAPDH were used as housekeeping genes for normalization. Gel electrophoresis bands for Caspase 3 and β-actin were displayed for reference (Fig. 5A). Normalized expression of Caspase 3 indicated a non-statistically significant increase with peptide treated cells versus untreated samples (Fig. 5B). KI67 and Survivin gel bands were similarly displayed along with GAPDH, for treated and untreated cells (Supplementary Fig. 1A and 2A). Peptide treatment significantly decreased normalized expression of proliferation indicator KI67 (Supplementary Fig. 1B), while downregulating Survivin expression as a caspase pathway inhibitor.

### Protein expression

The expression levels of PARP-1 were examined in treated and untreated U2OS cells, as the protein is cleaved by various caspase enzymes and is expressed separately once cleaved as a smaller size protein on the membrane (Fig. 5C) [37]. Expression data was presented as the ratio of the full-length protein expression level to that of the cleaved portion of the protein, normalized to endogenous control data (Fig. 5D). A high level of expression ratio indicates higher expression of full-length PARP-1 compared to the cleaved variant; low expression levels were associated with more cleavage protein compared to full-length expression. Our data suggests that treatment with IC50 causes cells to have decreased expression in the ratio of full-length protein to cleaved PARP-1 protein.

### Disk diffusion assay

**Fig. 6:**
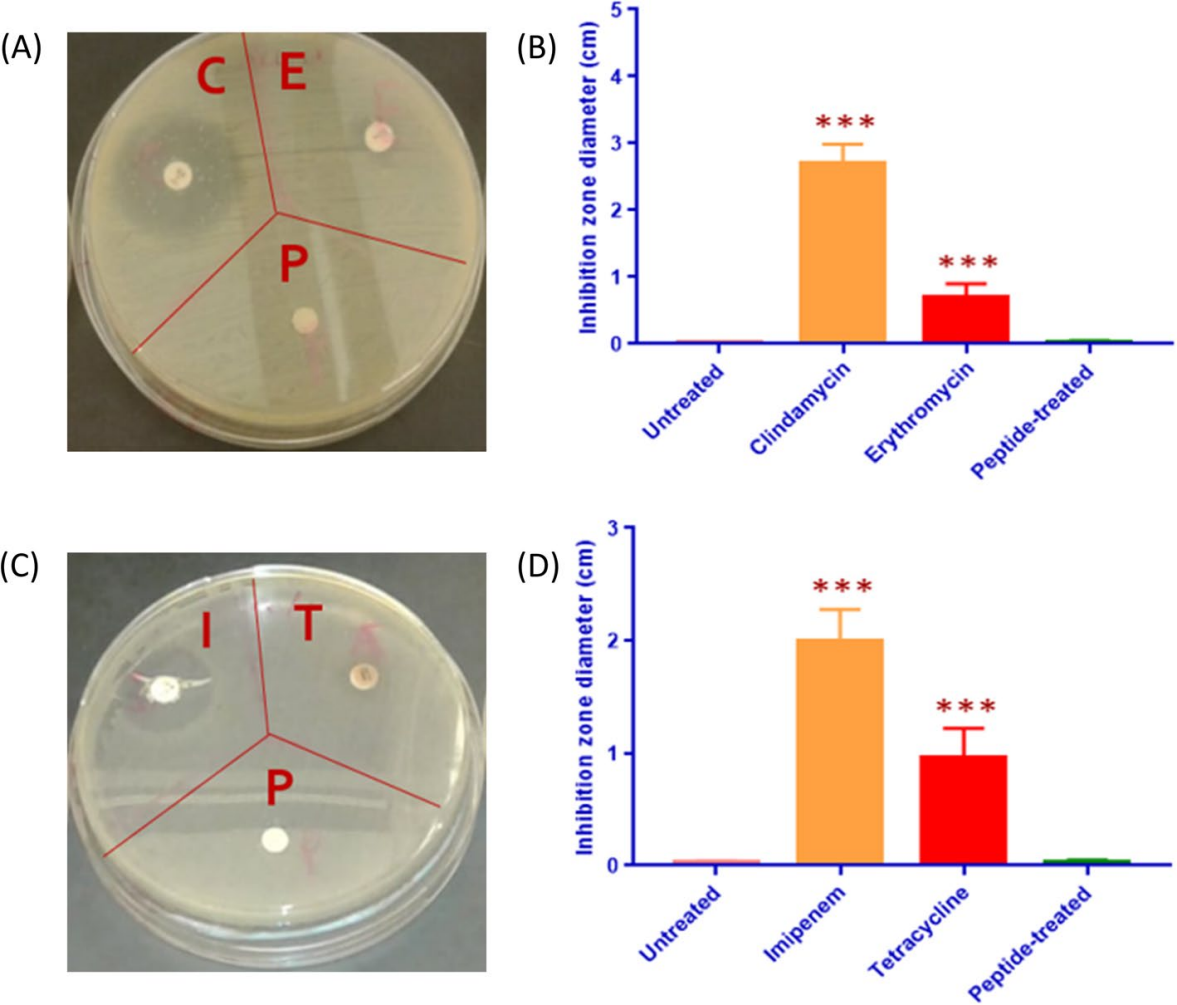
Susceptibility of *S. aureus* and *E. coli* to the peptide. **A** *S. aureus* representative plate images of inhibition zones generated by clindamycin [C], erythromycin [E], and peptide [P] disks following 24 h inverted incubation at 37 °C. **B** Average inhibition zone diameters (cm) caused by the antibiotics and the IC50 concentration of the peptide on *S. aureus* cells. Inhibition zones were not observed in peptide treated disks, contrasting with statistically larger [C] and [E] zones. **C** *E. coli* representative plate images of inhibition zones generated by imipenem [I], tetracycline [T], and IC50 peptide [P] disks, following 24 h incubation at 37 °C. **D** Average diameter inhibition zones (cm) from antibiotic exposure and IC50 concentration of the peptide on *E. coli* spread plates. Inhibition zones were not observed around peptide disks, contrasting with antibiotic impregnated disks, where [I] and [T] showed significant inhibition zones

*S. aureus* plates containing disks impregnated with Clindamycin [C], and Erythromycin [E], and the IC50 concentration of the peptide [P] were etched in agar plates with overnight culture spread (Fig. 6A). Inhibition zone diameter was measured by subtracting the inhibition zone created by impregnated disks from the diameter of the filter paper disks themselves. Bacterial growth of *S. aureus* was not inhibited by [P]; compared to [C] and [E] which left a large (approx. 2.84 cm) and a smaller (approx. 0.67 cm) inhibition zone diameter, respectively (Fig. 6B). *E. coli* grown plates were etched with disks soaked in peptide and antibiotics Imipenem [I] and Tetracycline [T] (Fig. 6C). Peptide-impregnated disks did not show any detectable inhibition zone, however, [I] and [T] antibiotics caused significant inhibition, approx. 1.98 cm and 0.95 cm diameter, respectively (Fig. 6D).

### Bacterial cell viability and antimicrobial activity

**Fig. 7:**
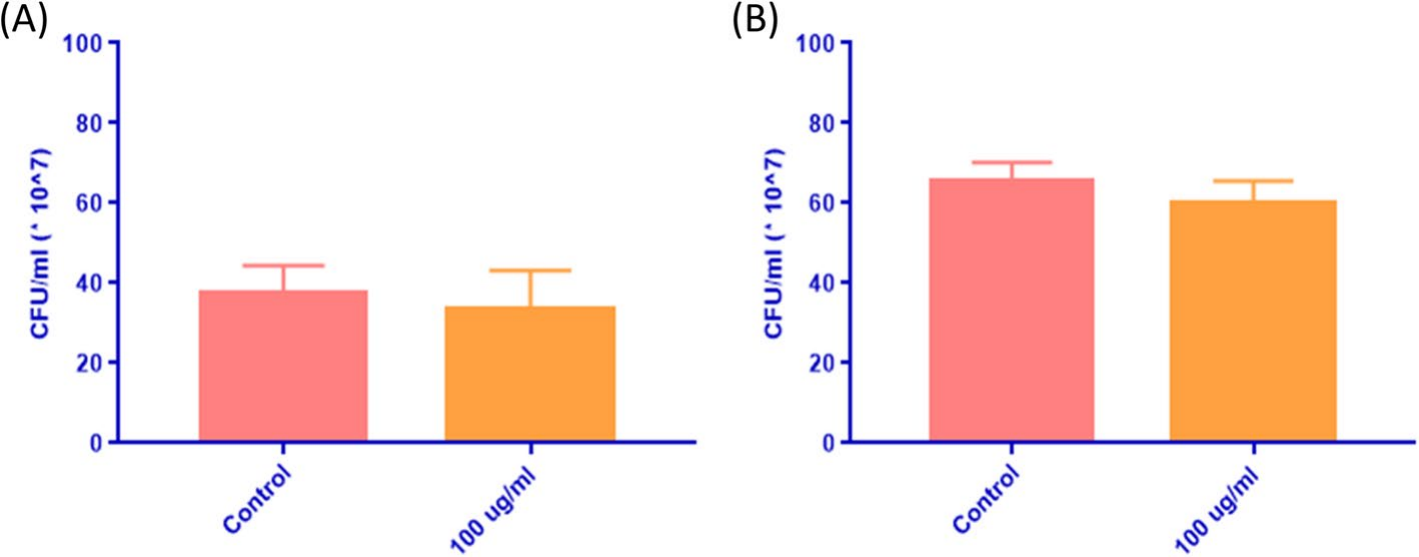
Effect of the IC50 concentration of the peptide on *S. aureus* and *E. coli* cells. **A** The graph shows the average number of CFUs/ml in *S. aureus* plates from untreated plates (pink bar) and treated plates with the IC50 concentration of the peptide (orange bar). **B** Average CFUs/ml in untreated *E. coli* plates vs. treated with the IC50 concentration of the peptide

Viable colony count was used to evaluate the effect of the peptide IC50 on *S. aureus* and *E. coli*. Addition of IC50 concentration of the peptide to cultures of *S. aureus* and *E. coli* did not result in any significant reduction of the CFU/ml compared to those from the untreated control (Fig. 7A and B).

### Scanning Electron Microscopy Assay (SEM)

Cellular and colony morphology of peptide-treated *S. aureus* and *E. coli* cells were detected via an LEO Supra 55 FE-SEM. Both *S. aureus* peptide-treated and untreated samples showed similar colony morphology at lower magnification (1000x – 1790x), where images for both strains displayed small-moderate sized colonies, with a transition from monolayered to multilayered growth and 3D structures (Supplementary Fig. 3 A1 and C1). Upon closer observation at higher magnification (2000x – 7760x), cells were observed to be surrounded with debris particles (Supplementary Fig. 3 A2 and C2), which was distinct from ampicillin-treated samples, where formation of single layered colonies was identified. Increased presence of scattered cell debris was also featured in low and high magnification images (Supplementary Fig. 3 B1 and B2). *E. coli* samples revealed a clear distinction between peptide and ampicillin treated samples, where a large cluster of moderate sized colonies (10 µm) were identified in the former, while absence of colonies and high presence of cell debris aggregates was observed in the latter (Supplementary Fig. 4 A1 and B1). Similar observations were detected at higher magnification (Supplementary Fig. 4 A2 and B2).

### Hemolytic activity assay

**Fig. 8:**
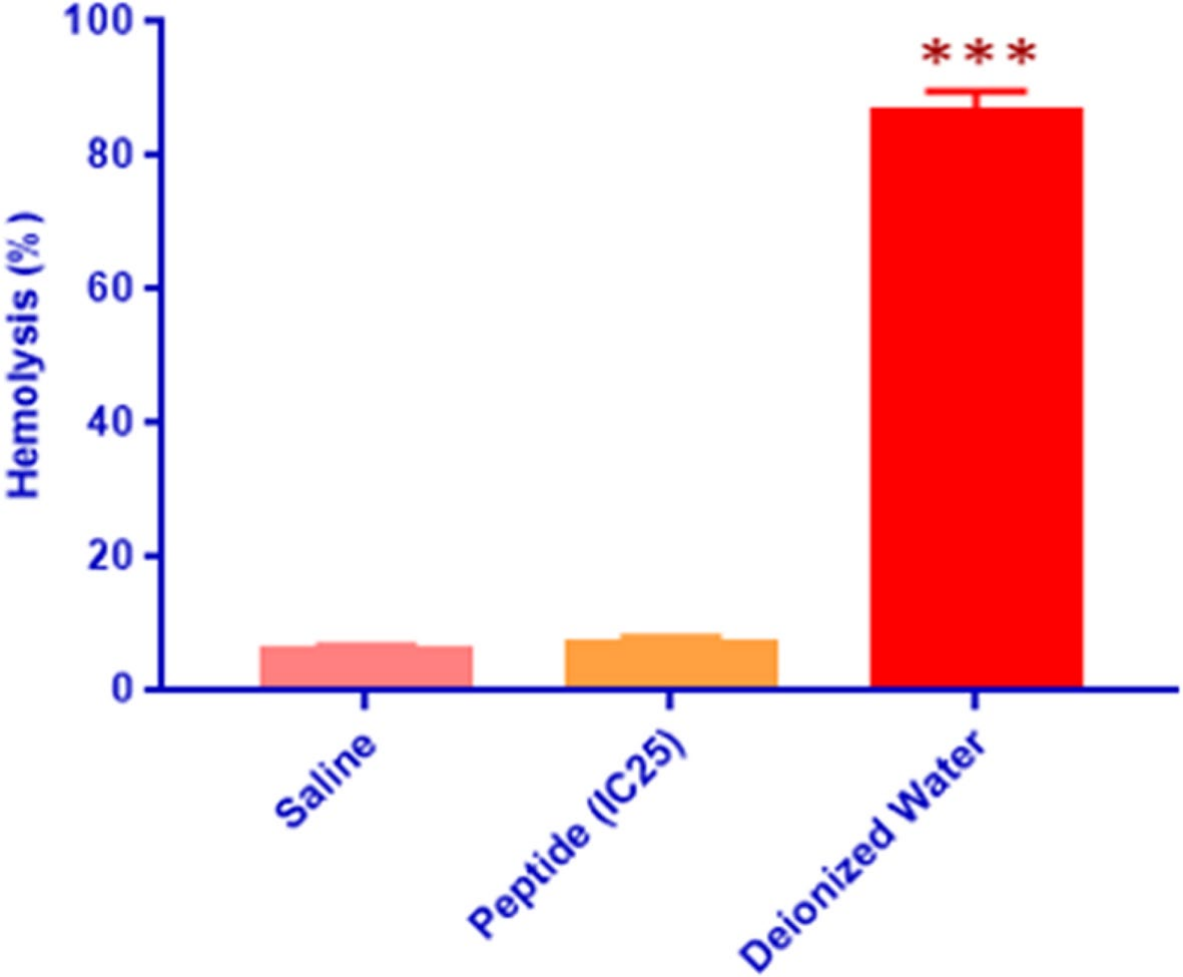
Effect of the peptide on red blood cells (RBCs). Average hemolysis percentage of saline, IC25 concentration of the peptide on RBCs, and distilled water following one hour-treatment of RBCs. Peptide treatment did not have an effect on hemolysis, compared to saline, while deionized water excessively ruptured RBCs with 1 h of exposure

The effect of peptide treatment on human red blood cells (RBCs) was examined to validate in-silico analysis that indicated relatively stability for blood-borne half lifetime. The average hemolytic percentages for both the peptide-treated and the saline-treated samples were statistically similar (Fig. 8). Distilled water-treated cells showed near complete hemolysis after 1 h of exposure.

## Discussion

We developed a workflow to identify a list of potential ACPs derived from the Red Sea metagenomics library, a novel and unexplored peptide dataset, selecting the candidate(s?) based on highest scores for cationicity, amphipathicity, stability, and homeodomain size. We subsequently moved to predict structure and functional validation of the potential anticancer activity. The peptide showed significant alignment with multiple bacteriocins, a family of bacterial cationic peptide toxins with antimicrobic activity, specifically Azurin like bacteriocins that have lately shown potent cytotoxic and apoptosis-inducing effects in breast cancer and skin cancer cells [24, 38–40]. The peptide structure was predicted to have two helical chains covering more than 60% of its sequence, characteristic of ACPs essential for their interaction with the cancer cell membrane [2, 14]. The peptide’s half-life was predicted to be around 1 s in an intestinal environment, indicative of its susceptibility to proteolytic degradation after systemic administration. However, the blood-borne half-life was predicted to be significantly higher, lasting 22.9 min, which indicates its relative stability intravenously [25]. Yet, experimentally established peptides have shown blood-infusion half-lives, varying from 2 h – 6 days depending on size, and application [41, 42]. Peptide stability should be further assessed to verify kinetic profile and biodegradation output via incubation with biological matrices such as blood, serum, gastric, or intestinal fluid [43]. We subsequently performed an alignment test on the peptide sequence to identify the homeodomain superfamily and modeled its structure against PDB, giving more credence to our results. Moreover, the COACH webserver helped predict a DNA ligand binding site, validated for the specific homeodomain family that was attributed to the peptide. The COFACTOR database also helped us annotate its biological function to potentially mimic another Antp, a regulatory homeodomain in Drosophila, with penetrative intracellular capabilities [33].

We screened U2OS osteosarcoma cells for 24 h with increasing concentrations of peptide and found at low concentrations (1 & 10 µg/ml) no change in viability. However, from 100 µg/ml and every concentration higher, cells displayed a progressive pattern with increase in cytotoxicity. Normal L929 mouse fibroblasts were also examined for selectivity towards the peptide, where these findings were used to establish a selectivity index (SI) defined as the IC50 value of the normal cell line and dividing by the IC50 of the cancer cell line. However, the IC50 value of L929s was out of the scope of tested concentrations and thus an SI could not be calculated. Additionally, the low hemolytic percentage for the peptide-treated sample was sufficiently significant to indicate that no reactivity of the peptide with RBCs was observed and these normal cells were unaffected by peptide exposure. Similar helical and amphipathic peptides have shown a propensity to initiate apoptotic programmed cell death [44, 45]. At low concentrations, temporin-1CEa lost structural integrity at the cell surface level, and at higher concentrations had accumulated beyond a particular threshold that led to infiltration of the cellular and nuclear membranes to initiate apoptosis. Thus, the need for examination of peptide treatment for different osteosarcoma cell lines, such as SAOS-2, and other cell lines of different cancer types becomes essential to elucidate a wider profile of cytotoxicity. An important factor not interrogated in our work is the duration of peptide exposure, where U2OS cells were only exposed to the peptide for a short 24 h period. Examples of two different peptides (HNP-1 & Lcfin-B) with two varying effective exposure durations and differing modes of action. Peptides causing membrane disassembly showed faster cytotoxicity than those causing DNA damage or synthesis inhibition. Additionally, necrotic peptides were found to expedite cell death, compared to those progressing in the apoptotic pathway. Thus, increased exposure may provide insight into a more accurate assessment of the peptide’s mode of action.

Our observations of U2OS morphological changes from peptide treatment with 100 µg/ml showed an evident shrinkage of cells while maintaining cytoplasm architecture. Increasing the concentration to 300 µg/ml caused a change in membrane structure with rupturing occurring in significantly more cells. At 500 µg/ml, cells were either lysed, or formed a circular morphology. These changes are consistent with apoptotic death events in which shrinkage and membrane disintegration are the main key events of the pathway [46]. We also found the migratory capacity of U2OS cells in scratch-wound healing assay to be significantly debilitated following IC50 peptide exposure. The complementary decrease in KI67 gene expression was an indicator that peptide treatment resulted in mitigation of proliferation capacity by cells. Deeper gene expression analysis into the MAPK/ERK signaling pathway through p38 MAPK and ERK 1/2 is required to elucidate the implications of peptide treatment on cell migration and motility [47, 48].

We also assessed the mode of cell death cause by the peptide through Annexin V assay, with a high affinity to bind to Phosphatidyl serine (PS) externalized on the surface of early apoptotic cells and bound to Alexa Fluor 488. The red fluorescent DNA binding PI did not penetrate live or early apoptotic cells and instead was bound tightly to DNA of necrotic and late apoptotic cells with ruptured membranes. Annexin stained untreated cells showed few necrotic/apoptotic events, compared to peptide treated cells, where most cells were stained green, an indication they underwent an apoptotic pathway. Higher magnification microscopy highlighted ghost aggregates in cells that were not stained with either Annexin or PI, with disintegrated budding membranes from apoptotic bodies. These observations supported an interim condition towards an apoptotic mode of action [49, 50]. The morphological changes depicted in our findings suggested the lack of necrotic cell swelling, membrane blebbing and cellular rupture, thus discounting the effect of necrosis as a possible mechanism of cell death. However, electron microscope based morphological analysis of apoptosis related nuclear and mitochondrial changes is required for a more comprehensive understanding of the mode of cell death caused by the peptide. Mitochondrial transmembrane potential and ATP level should be assessed to evaluate the implications of peptide exposure on metabolic function and potential for cancer cell recovery from short-term peptide administration [51, 52]. A fluorescently labeled peptide conjugate will provide insight into the mechanistic approaches by which the peptide operates, and its deposition within various sections of the cell.

Gene and protein expression for Caspase 3, KI67, Survivin and PARP-1 support the theory of the peptide instigating apoptotic mode of action. KI67 and Survivin both showed decreased gene expression levels, however, Caspase 3 specifically exhibited a non-statistically significant change, an indicator into the complex molecular nature of apoptotic pathways and the need for further investigation into elucidating the extent of caspase inhibition. DNA laddering and expression of other effector caspase apoptotic markers such as Bcl2, BAX, APAF-1 and other IAPs, are to be evaluated for further proof of apoptotic action [53, 54]. The protein expression of PARP-1 full length protein ratio to cleaved expression decreased as a function of peptide screening. PARP-1 acts to repair DNA double-strand breaks through the microhomology-mediated end joint (MMEJ) pathway and is highly upregulated in the incidence of cancer [55, 56]. Thus, our findings with PARP-1 are promising, however require further validation through the assessment of associated MMEJ repair proteins such as BRCA, FEN1, and XRCC1 [57, 58]. Additionally, other peptides were found to mediate the cytotoxic effect in a caspase dependent manner at certain concentrations, and in a caspase independent manner with a change in these concentrations [7, 43, 59]. Therefore, expression analysis should encompass multiple dose dependent responses and exposure periods, for different cancer cell lines.

Assessment of the antibacterial effect of the peptide represented an important component in ruling out any possible effect on model bacterial species of the human microbiome [30]. The latter play essential roles in food digestion, immune system regulation, vitamins production, and protection against infections [60], thus the preservation of the microbiome is vital during and after chronic exposure to the peptide. Avoiding the disruption of the human microbiome is important as interference may cause microbiome dysbiosis, which may incur diseases such as, inflammatory bowel disease, type 1 diabetes mellites, colorectal cancer, and allergenic responses [61]. *E. coli* and *S. aureus* bacterial species were selected as they are both extensively studied human commensals and as good model organisms for Gram-negative and Gram-positive bacteria, respectively [62]. In addition, these species are among the most abundantly found in the human microbiome. *E. coli* is copiously present in the human gastrointestinal tract, while *S. aureus* grows on human skin and in the nasal tract, forming an essential component in regulating digestion and maintaining immune function, respectively [63].

We further investigated the antibacterial activity of the peptide by evaluating the viable cell count, disk diffusion assay, and scanning electron microscopy (SEM). These assays collectively assessed the effect of the peptide on the morphology, viability, and growth of *E. coli* and *S. aureus* bacterial cells, with IC50 peptide treatment. The viable count of *E. coli* and *S. aureus* was minimally affected with peptide incubation, and in accordance with reported *in-silico* analysis. This was supported by computational data, identifying multiple coiled regions constituting more than 60% of the peptides sequence, suggesting it possessed low antimicrobial activity [64]. Peptides exhibiting a high degree of coiling were observed to have low antimicrobial activity, due to reduced accessibility while interfacing with bacterial cells [65]. The AMPA findings suggested that regions 1–6 and 12–16 were the only two sections that possessed a relatively high antimicrobial activity, which represented 32% of the entire peptide sequence. Accordingly, the composition of these two regions can be further modified to reduce the antimicrobial activity of the peptide, while preserving its anticancer properties [66]. There was no significant change in the number of viable bacterial colonies before and after treatment. Additionally, SEM microscopy corroborated these results by highlighting no change in morphology with peptide treatment. These findings suggest the peptide’s safety towards *E*. *coli* and *S. aureus* strains. Thus, expanding the umbrella of testing the peptide to other bacterial species and other cancer cell lines will loan insight into further expanding the peptide’s application and aid in understanding the mechanism of action by which apoptosis occurs.

## Conclusion

We have developed a 34-mer cationic, amphipathic cytotoxic peptide with the potential to be further developed into an ACP drug. The peptide was selected with an *in-silico* sequence and structural similarity to homeoproteins. Its cytotoxicity was hypothesized to be attributed to a non-specific selective membranolytic effect on cancer cells, as a cationic helical and amphipathic peptide. Osteosarcoma cells were critically impacted by treatment with the peptide in a dose dependent response, while L929 mouse fibroblasts did not respond to treatment, thus the peptide displayed relative selectivity between the two cell lines. Combined migration/proliferation of cells was reduced in cells exposed to peptide, as observed from scratch-wound healing assay. Mode of cell death was indicative of apoptotic action as per increased staining of annexin V in treated cells and the minor gene expression increase in Capsase 3, the down regulation of KI67 and Survivin, in addition to attenuated PARP-1 protein expression. No definitive pathway could be highlighted from these findings alone, but apoptosis is the leading effector. The non-statistically significant overexpression of Caspase 3 does not disprove our hypothesis since some apoptosis mechanisms are caspase independent (parthanatos) associated with PARP. The peptide exhibited a minimal antimicrobial activity on *E. coli* and *S. aureus* bacterial strains, suggesting its safety on the microbiological level and that it may not disrupt the microbiome constituting these species. To improve the computational predictive model, enzymatic interference, peptide docking mechanism, and environmental conformation changes allow for the determination of peptide stability and preservation. Establishing the peptide as a broad ranging ACP treatment requires further screening of a panel of cancer cell lines at multiple durations, while definitive identification of the mode of cell death required electron microscopy of the cell membrane and morphological whole cell imaging, in addition to gene and protein expression analysis of apoptosis and necrosis pathway markers.

### Supplementary Information

**Additional file 1: Supplementary Figure 1.** Gene expression of KI67 in untreated vs peptide treated U2OS cells. Untreated and treated samples of GAPDH and Caspase 3 primers were run on an agarose (1.5%) gel. Original unedited image of gel can be found in supplementary figure 7. (A) Expression of KI67 and GAPDH (indigenous control) in treated and untreated cells through gel electrophoresis. (B) Normalized expression of KI67 in untreated and treated cells (*** *P*< 0.0001, *n*=4). **Supplementary Figure 2.** Gene expression of Survivin in U2OS cells. Untreated and treated samples of GAPDH and Survivin primers were run on an agarose (1.5%) gel. Original unedited image of gel can be found in supplementary figure 7.  (A) Gel electrophoresis Survivin and GAPDH (indigenous control) gene expression in untreated and peptide treated in cells. (B) Normalized expression of Survivin in untreated and treated cells. Treatment appeared to significantly decrease expression of Survivin (*** *P*< 0.0001, *n*=4). **Supplementary Figure 3.** S. aureus cells under Scanning Electron Microscopy at two magnification powers. (A1) Peptide-treated cells at low magnification level. (B1) Ampicillin-treated cells at low magnification level. (C1) Control cells at low magnification power. (A2) Peptide-treated cells at higher magnification level. (B2) Ampicillin-treated cells at higher magnification level. (C2) Control cells at higher magnification power. **Supplementary Figure 4.** E. coli cells under Scanning Electron Microscopy at two magnification powers. (A1) Peptide-treated cells at low magnification level. (B1) Ampicillin-treated cells at low magnification level. (A2) Peptide-treated cells at higher magnification level. (B2) Ampicillin-treated cells at higher magnification level. (C) Control cells. **Supplementary Figure 5.** Agarose gel (1.5%) electrophoresis of untreated and treated samples with Caspase 3 and B-actin primer gene expression. **Supplementary Figure 6.** Nitrocellulose membrane of PARP-1 cleaved, PARP-1 uncleaved, and GAPDH protein expression. (A) Captured image of membrane via transilluminator. (B) White tray image of membrane expression. (C) Captured image of membrane with black tray background. **Supplementary Figure 7.** Agarose gel electrophoresis of untreated and treated samples with KI67, Survivin, B-actin primer gene expression. (A) Ethidium bromide UV image of gels with samples expressing KI67 and Survivin primers (B) Gel images of GAPDH expression of untreated cells. 

## Data Availability

The methods utilized for the broad scanning of anticancer peptide activity from the AUC/KAUST Red Sea Microbiome Bioroject dataset (PRJNA299097) and (PRJNA193416) was adapted from [18], and implemented based on results produced from [17], where the same accession numbers of experimentally proven anticancer peptides from antimicrobial databases APD3 (https://aps.unmc.edu/database/anti), CAMP (http://www.bicnirrh.res.in/antimicrobial), and DADP (http://split4.pmfst.hr/dadp/) were compared to another set of antimicrobial peptides (AMPs) with no anticancer activity. The experimental data used to support the findings of the in-silico predications in this study are included in the article and in the supplementary material. Inquiries regarding raw data can be made through the corresponding author, Asma Amleh; Email: aamleh@aucegypt.edu.
