# Supplementary material for: Derivation of a novel antimicrobial peptide from the Red Sea Brine Pools modified to enhance its anticancer activity against U2OS cells
Source: BMC Biotechnol. 2024 Mar 15;24:14. doi: 10.1186/s12896-024-00835-8 (PMC10943910; doi:10.1186/s12896-024-00835-8)
Supplement: Supplementary file 1 — Additional file 1: Supplementary Figure 1. Gene expression of KI67 in untreated vs peptide treated U2OS cells. Untreated and treated samples of GAPDH and Caspase 3 primers were run on an agarose (1.5%) gel. Original unedited image of gel can be found in supplementary figure 7. (A) Expression of KI67 and GAPDH (indigenous control) in treated and untreated cells through gel electrophoresis. (B) Normalized expression of KI67 in untreated and treated cells (*** P< 0.0001, n=4). Supplementary Figure 2. Gene expression of Survivin in U2OS cells. Untreated and treated samples of GAPDH and Survivin primers were run on an agarose (1.5%) gel. Original unedited image of gel can be found in supplementary figure 7. (A) Gel electrophoresis Survivin and GAPDH (indigenous control) gene expression in untreated and peptide treated in cells. (B) Normalized expression of Survivin in untreated and treated cells. Treatment appeared to significantly decrease expression of Survivin (*** P< 0.0001, n=4). Supplementary Figure 3. S. aureus cells under Scanning Electron Microscopy at two magnification powers. (A1) Peptide-treated cells at low magnification level. (B1) Ampicillin-treated cells at low magnification level. (C1) Control cells at low magnification power. (A2) Peptide-treated cells at higher magnification level. (B2) Ampicillin-treated cells at higher magnification level. (C2) Control cells at higher magnification power. Supplementary Figure 4. E. coli cells under Scanning Electron Microscopy at two magnification powers. (A1) Peptide-treated cells at low magnification level. (B1) Ampicillin-treated cells at low magnification level. (A2) Peptide-treated cells at higher magnification level. (B2) Ampicillin-treated cells at higher magnification level. (C) Control cells. Supplementary Figure 5. Agarose gel (1.5%) electrophoresis of untreated and treated samples with Caspase 3 and B-actin primer gene expression. Supplementary Figure 6. Nitrocellulose membrane of PARP-1 cleaved, PARP-1 un [file 12896_2024_835_MOESM1_ESM.pptx]

## Slide 1
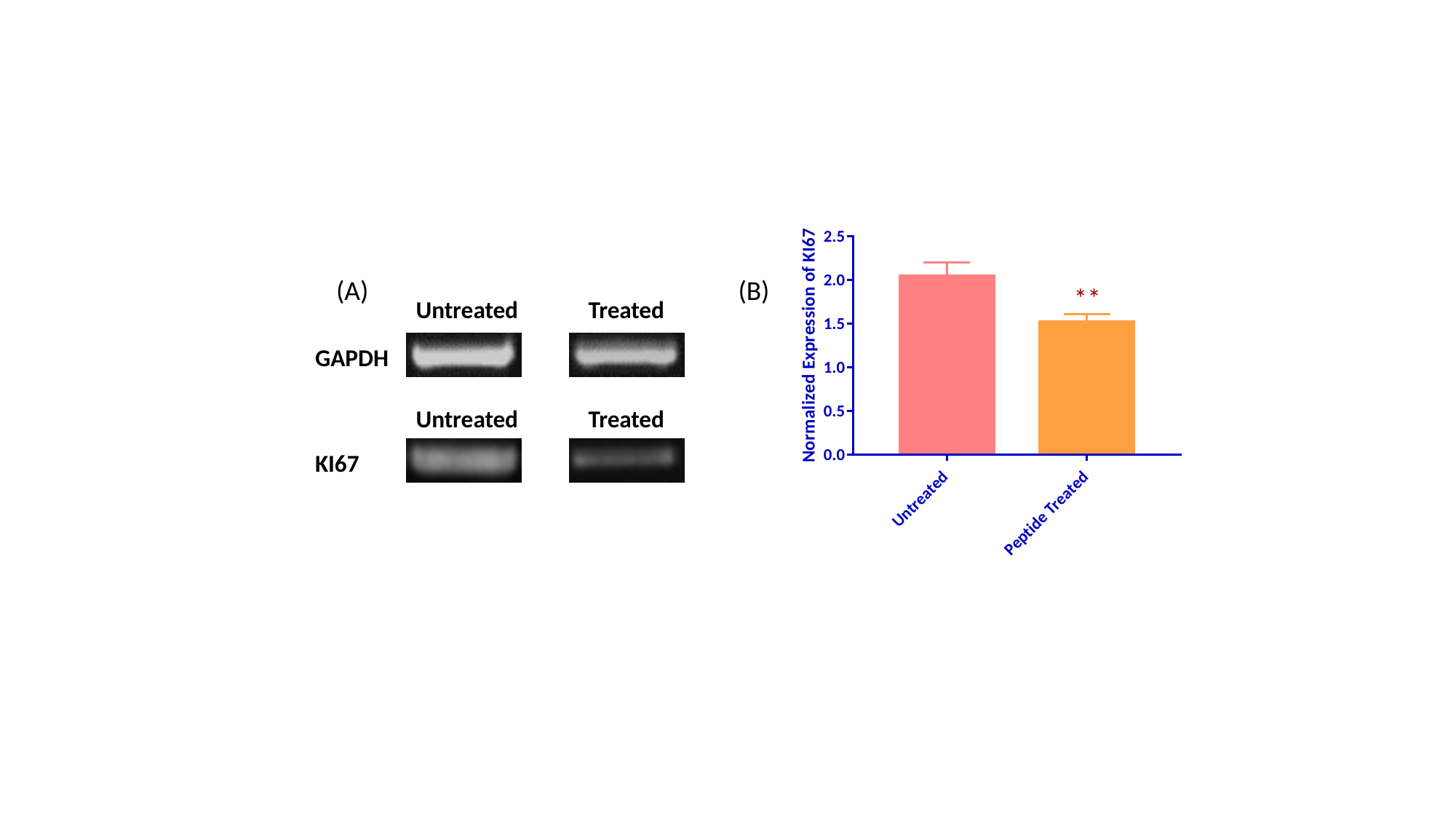

(A)
(B)
Untreated
Treated
GAPDH
Untreated
Treated
KI67

## Slide 2
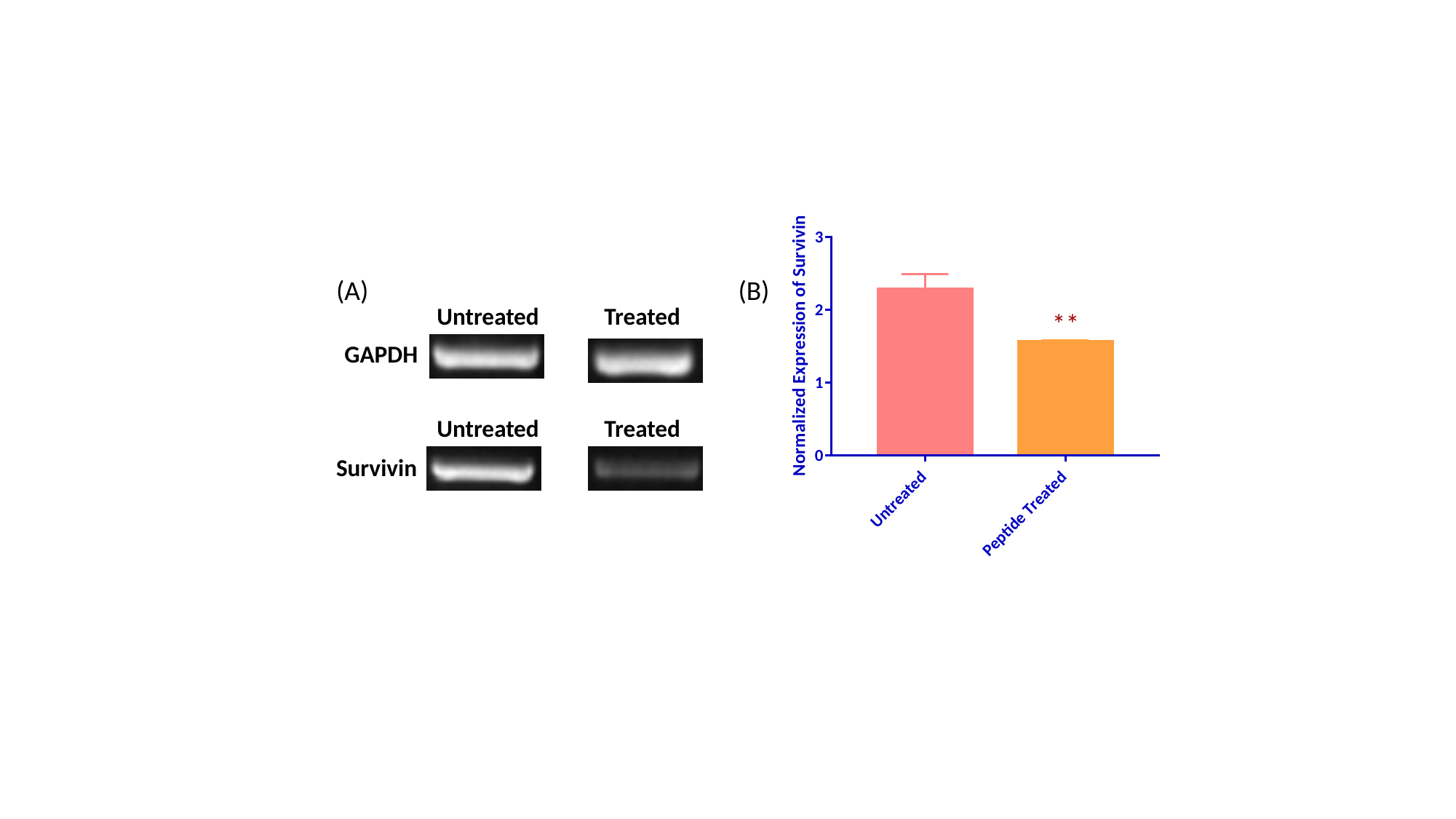

(A)
(B)
Untreated
Treated
GAPDH
Untreated
Treated
Survivin

## Slide 3
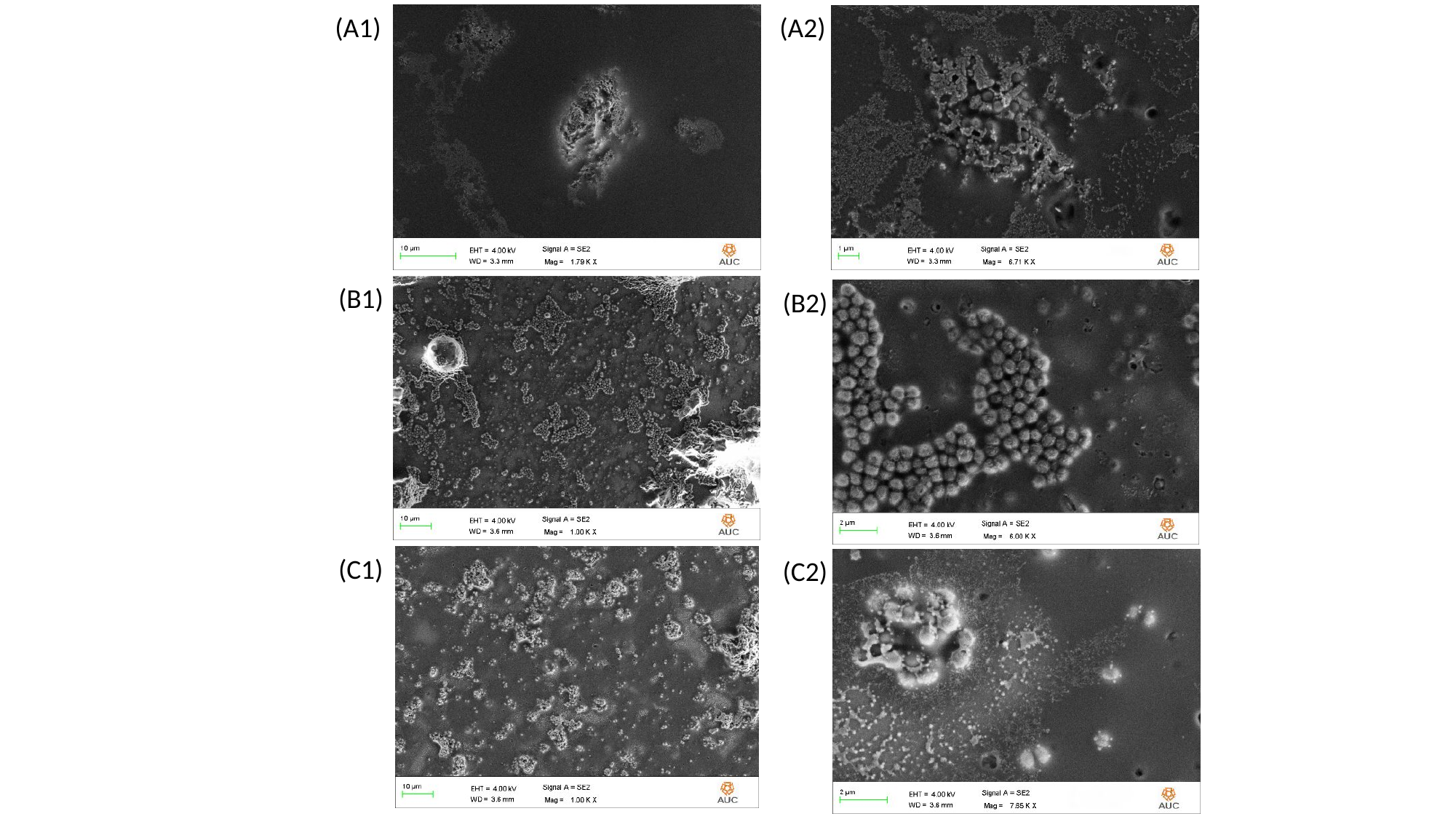

(A1)
(A2)
(B1)
(B2)
(C1)
(C2)

## Slide 4
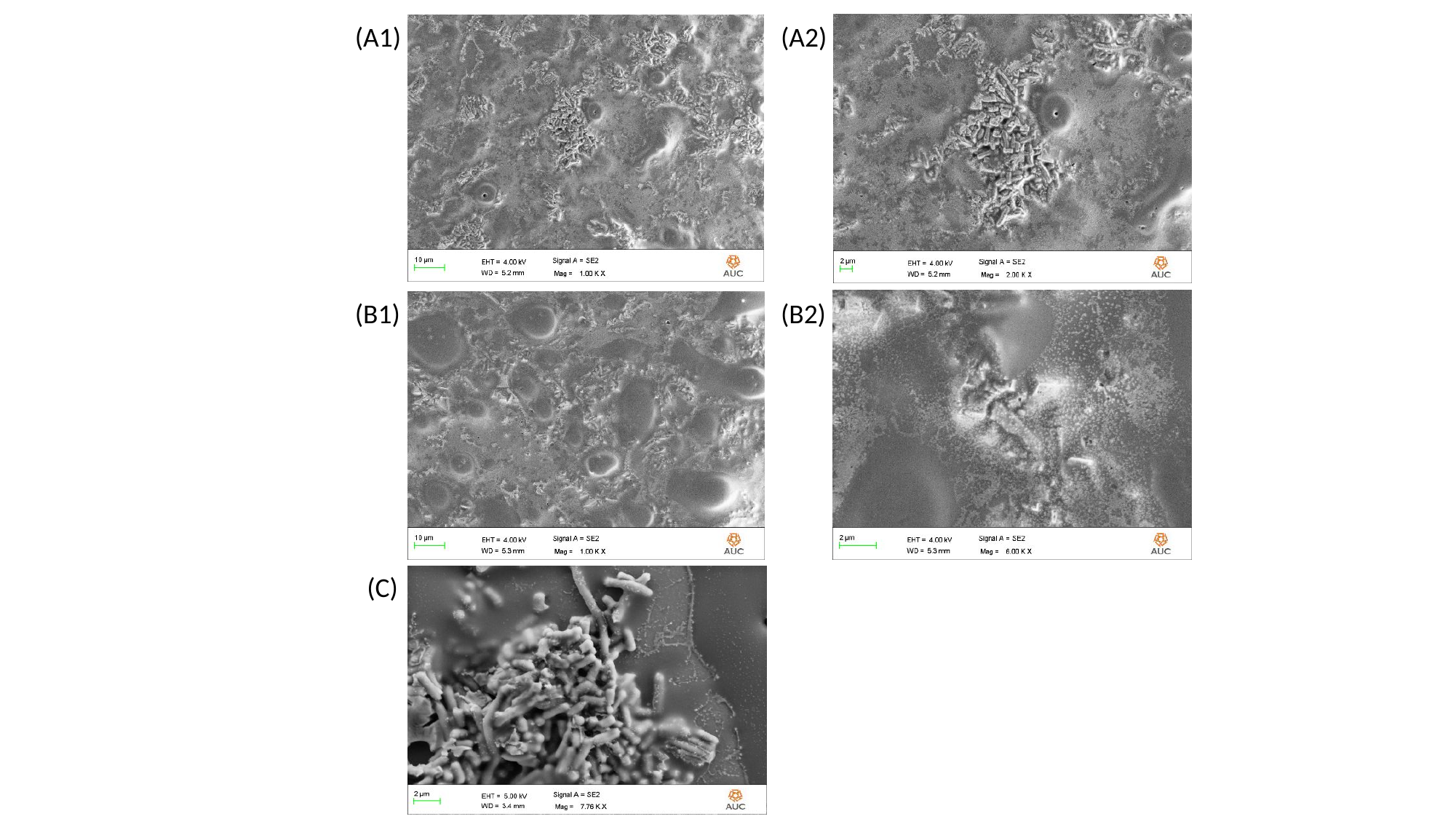

(A1)
(A2)
(B1)
(B2)
(C)

## Slide 5
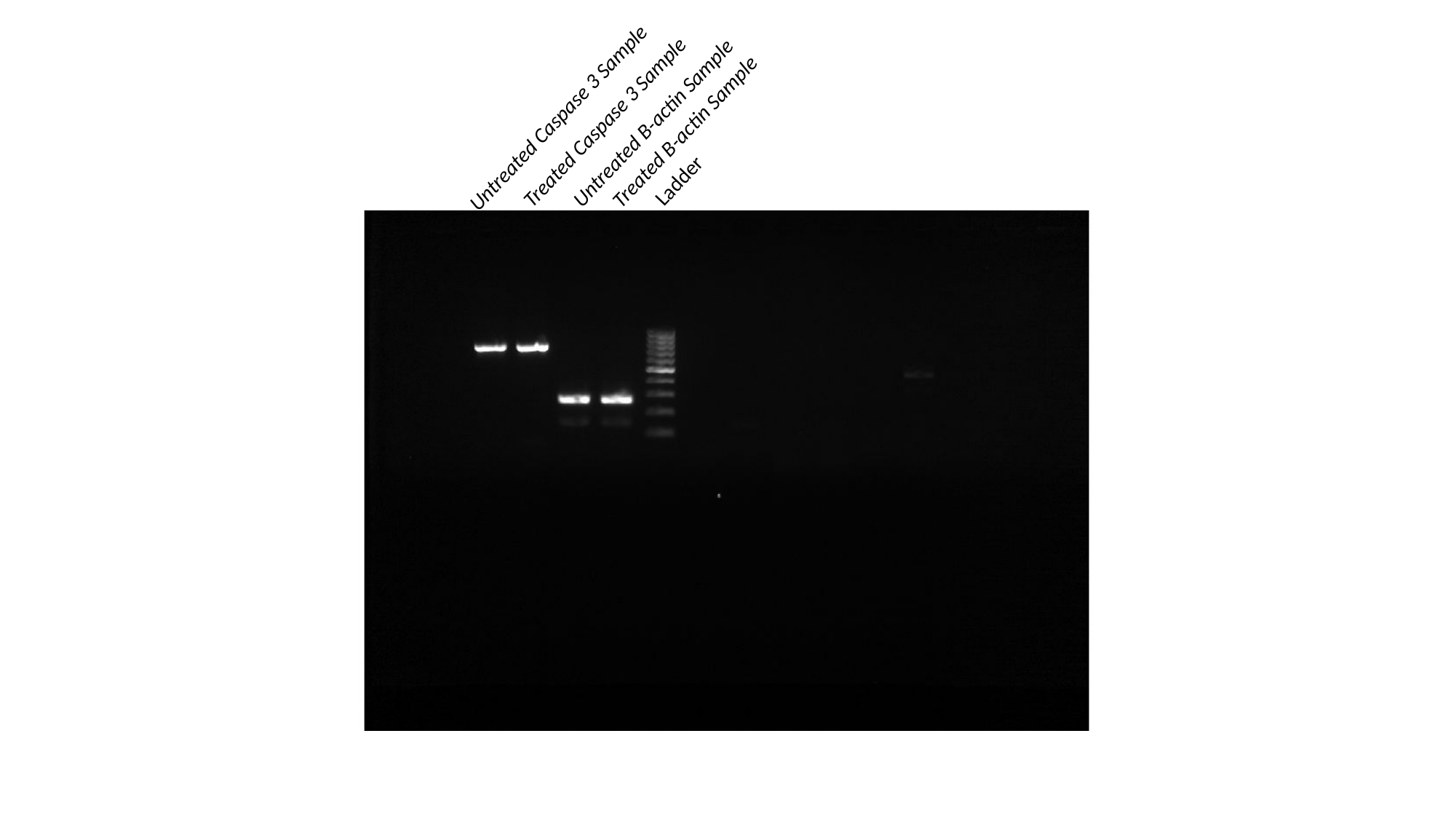

Untreated B-actin Sample
Treated B-actin Sample
Treated Caspase 3 Sample
Untreated Caspase 3 Sample
Ladder

## Slide 6
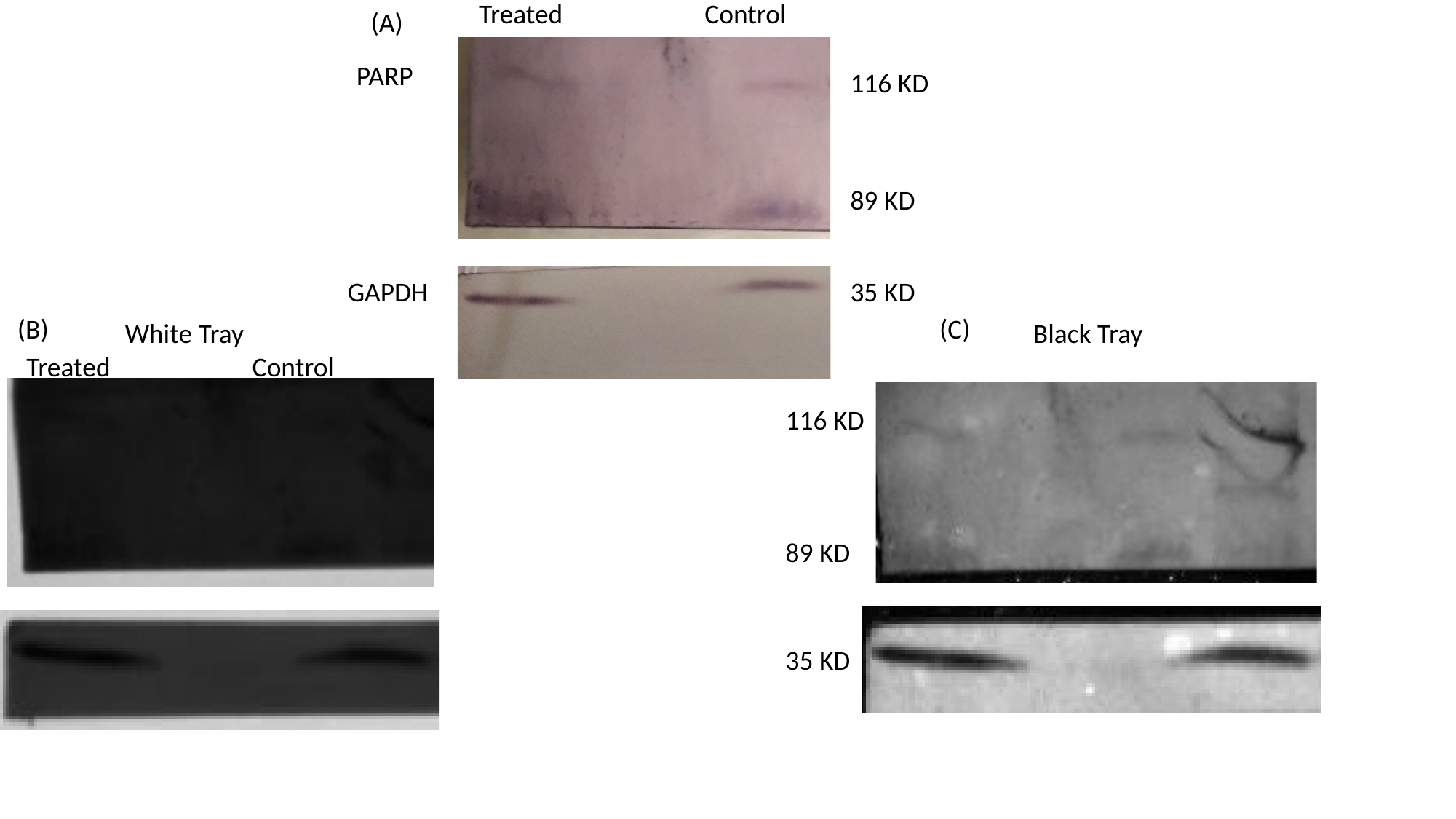

(A)
Treated Control
PARP
116 KD
89 KD
GAPDH
35 KD
(B)
(C)
White Tray
Black Tray
Treated Control
116 KD
89 KD
35 KD

## Slide 7
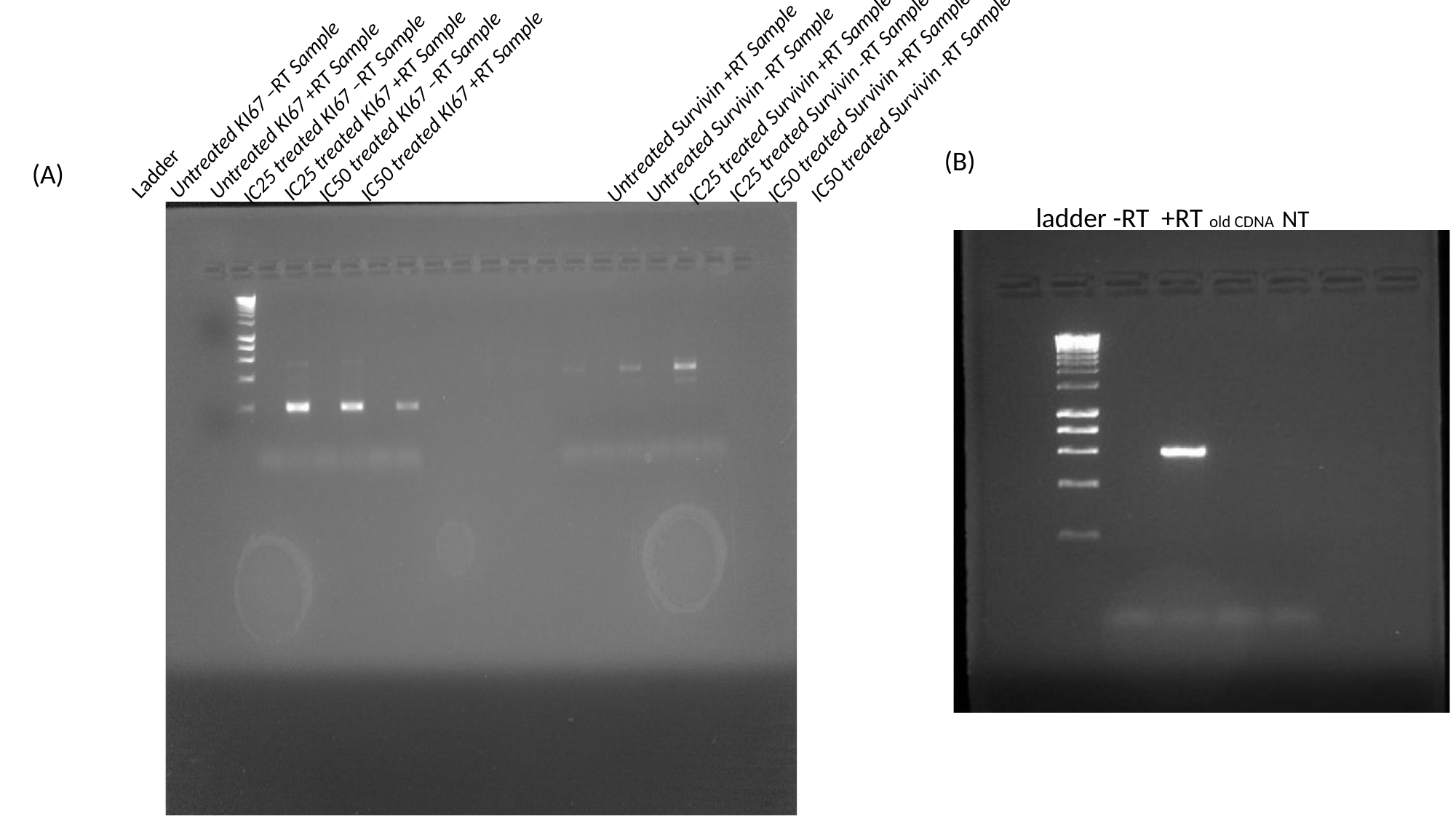

IC25 treated KI67 +RT Sample
IC50 treated KI67 +RT Sample
IC25 treated Survivin -RT Sample
IC50 treated Survivin -RT Sample
IC50 treated KI67 –RT Sample
IC50 treated Survivin +RT Sample
IC25 treated KI67 –RT Sample
IC25 treated Survivin +RT Sample
Untreated KI67 –RT Sample
Untreated KI67 +RT Sample
Untreated Survivin -RT Sample
Untreated Survivin +RT Sample
Ladder
(B)
(A)
 ladder -RT +RT old CDNA NT
